# Towards mapping the 3D genome through high speed single-molecule tracking of functional transcription factors in single living cells

**DOI:** 10.1016/j.ymeth.2019.06.021

**Published:** 2020-01-01

**Authors:** Adam J.M. Wollman, Erik G. Hedlund, Sviatlana Shashkova, Mark C. Leake

**Affiliations:** Biological Physical Science Institute, Departments of Physics and Biology, University of York, YO10 5DD York, UK

**Keywords:** EMCCD, electron multiplying charge-coupled device, FISH, fluorescence *in situ* hybridization, GFP, green fluorescent protein, MSD, mean square displacement, PBS, phosphate-buffered saline, PSF, point spread function, RT, room temperature, ChIP-Seq, chromatin immunoprecipitation-sequencing, Yeast genome, Transcription factors, Single-molecule, Transcription, Gene regulation, Super-resolution

## Abstract

How genomic DNA is organized in the nucleus is a long-standing question. We describe a single-molecule bioimaging method utilizing super-localization precision coupled to fully quantitative image analysis tools, towards determining snapshots of parts of the 3D genome architecture of model eukaryote budding yeast *Saccharomyces cerevisiae* with exceptional millisecond time resolution. We employ astigmatism imaging to enable robust extraction of 3D position data on genomically encoded fluorescent protein reporters that bind to DNA. Our relatively straightforward method enables snippets of 3D architectures of likely single genome conformations to be resolved captured via DNA-sequence specific binding proteins in single functional living cells.

## Highlights

• Single-molecule imaging to determine genome organization in live budding yeast.
• Utilize astigmatism imaging to enable extraction of 3D position data.
• Use a transcription factor Mig1 to mark the genome at target genes.
• Map out 3D coordinates of target genes with millisecond sampling.
• Observe differences to 3C structure indicating dynamic heterogeneity of the genome.

## 1 Introduction

Variations in 3D genome architecture contribute to a large number of disorders, including autism, schizophrenia, congenital heart disease and cancer [1]. However, our knowledge of the functional organization of dynamic DNA in the complex, crowded physiological milieu of living cells remains limited. New methods to elucidate 3D genome structure may be valuable in improving our understanding of not only the native genomic architecture in normal cells but also of the development and progression of diseases associated with DNA structural abnormalities.

There are various existing tools to study 3D genome configuration, such as probing RNA-chromatin interactions, chromosome conformation capture (3C) techniques and microscopy-based approaches, including the 3C variant Hi-C that extends the capability of the technology by identifying longer range interactions across the whole genome [2], [3]. However, none of these methods are comprehensive on their own in regards to generating data representing a dynamic structure of an individual genome conformation from single, functional, living cells [4]. For example, 3C variant techniques are genome-wide, but the results represent the ensemble average of all genome configurations, and so lose dynamic information. Moreover, these methods cannot be performed *in vivo* and, furthermore, are population level techniques generating information from often several thousands of cells and so struggle to render important information concerning cell-to-cell variability, arguably a key feature in ensuring cell survival during conditions of high stress. Standard fluorescence *in situ* hybridization, FISH, is a traditional microscopy-based approach, which is widely used in DNA localization studies. 3D-FISH in combination with confocal microscopy and image reconstruction enables the analysis of the spatial arrangement of chromosomes. However, this technique, in its traditional form at least, requires sample fixation [5], and thus fails to render information concerning structural fluctuations in the genome with time. Recent advances in single-molecule fluorescence microscopy have provided fundamental insights into the interactions of proteins with DNA upon gene regulation in both prokaryotes and eukaryotes [3], [4]. Studies on live cells from a range of different species show that several types of proteins which bind to DNA, including those involved in chromatin remodeling, DNA replication, transcription and repair, operate as oligomeric clusters [6], [7], [8], [9].

Here we describe a novel approach for achieving 3D spatial resolution at millisecond time scales and single-molecule detection sensitivity directly in single living eukaryotic cells using astigmatism imaging [10]. We modified a method that generates a narrow field of laser illumination which produces high excitation intensities in the vicinity of single live cells [11], [12], [13], [14]. This technique is based on introducing astigmatism into the imaging path through insertion of a long focal length cylindrical lens between the microscope emission port and camera detector, which enables extraction of 3D spatial positions of single fluorescent reporter molecules. Astigmatism-based approaches allow imaging over an axial range comparable with the length scale of the nucleus in yeast cells. The method is also relatively easy and cheap to implement compared to competing techniques, such as multi focal plane imaging [15] and approaches which use helical shaped point spread function (PSF) imaging profiles [16]. Astigmatism imaging combined with Stochastic Optical Reconstruction Microscopy (STORM) has been used to image microtubules and clathrin coated pits in cells with spatial resolution which is an order of magnitude better than standard diffraction-limited optical resolution. However, STORM requires typically long imaging times so rapid dynamics are largely lost [17]. In a recent review of 3D imaging techniques, astigmatism imaging approaches perform well in lateral and axial resolution, as well as the axial range over which probes can be detected [18]. Multi focal plane imaging, most simply including biplane imaging, and double helix PSF microscopy, perform marginally better in regards to spatial resolution but these modalities are often complex and/or costly to implement, e.g. requiring multiple objective lenses and/or phase modulation optics. Recently, tilted light sheet microscopy combined with PSF engineering was able to map out the whole mammalian cell nuclear envelope [19] and may become a powerful future technique for 3D genome architecture. Besides optical advances, a novel experimental PSF-fitter software has been developed, which compensates for optical aberrations and enables 3D resolution even on setups without 3D optics [20]. However, to date, the software has not been used on living cells.

We utilize the budding yeast *Saccharomyces cerevisiae* and its DNA-binding Mig1 protein as a reference for genome mapping. Mig1 is a Zn-finger transcription factor which binds to target DNA sequences and under glucose-rich extracellular conditions represses expression of genes essential for metabolism of non-glucose carbon sources [21], [22]. In our previous work, we performed *in vivo* 2D Slimfield imaging of Mig1-GFP under glucose rich and depleted conditions. Our results indicated that Mig1 operates as 6–9-mer clusters, the main fraction of which, upon glucose repletion, is located in the nucleus and immobile. Glucose deprivation causes an increase of the clusters mobility and cytoplasmic import, however, a small portion of Mig1 was still detectable in the nucleus. We showed that immobile Mig1 molecules with apparent 2D diffusion coefficients lower than ∼0.1 µm^2^/s were likely to be bound to DNA, and that we could use 3C models combined with bioinformatics analysis to predict the likely Mig1 binding sites in 3D [7]. In our present work here, we directly image fluorescent Mig1 in 3D, and identify immobile Mig1 foci. We then compare our observations to the 3C model and provide valuable biological insights into the 3D eukaryotic genome architecture in single living cells. Our new method does *not* enable full 3D genomic architectures in yeast to be determined as a function of time, but rather enables snapshots of parts of the 3D genomic architecture to be resolved with exceptionally high time resolution.

## 2 Materials and methods

### 2.1 3D super-resolution single-molecule microscope

**Fig. 1:**
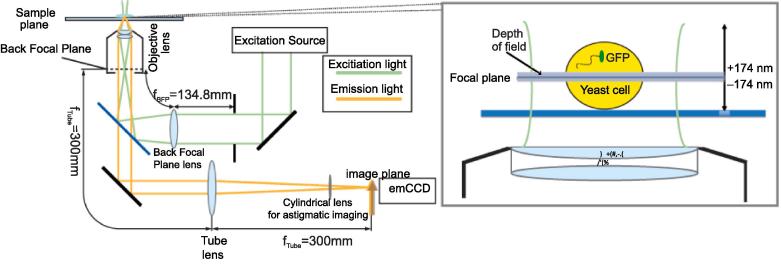
The astigmatism microscopy imaging system. The depth of field in a non-astigmatism microscope is indicated with the shaded band (zoom-in image on the right panel). The cylindrical lens is located just outside the imaging port of the microscope.

We constructed a bespoke astigmatism super-resolution fluorescence microscope, built around the body of a Nikon *Ti*-series epifluorescence microscope. A schematic of the optical design is shown in Fig. 1. We implemented Slimfield illumination to observe single GFP molecules in living cells, a method that generates high laser excitation intensities in the vicinity of single cells thereby enabling millisecond sampling [7]. Vortran 50 mW 473 nm and 561 nm wavelength lasers, coupled together using a dichroic mirror, were incident on a lens in a telescope with the objective lens to generate a collimated ∼20 µm (full width at half maximum) beam at the sample, with an intensity of typically 2.5–3 kW/cm^2^. The image was collected by a 300 mm focal length tube lens onto a Photometrics Evolve 512 Delta EMCCD camera, with a DV2 color splitter to enable separate, simultaneous imaging of GFP and mCherry fluorescent protein components in the sample. A cylindrical lens was placed between the tube lens and the camera for astigmatism imaging. The resulting magnification at the sample is 93 nm per pixel. The sample was held on a Mad City Labs XYZ positioning nanostage. Full details of filters and lenses in Supplementary Table 1.

### 2.2 Calibration of the microscope

#### 2.2.1 Fluorescent protein *in vitro* assay

**Fig. 2:**
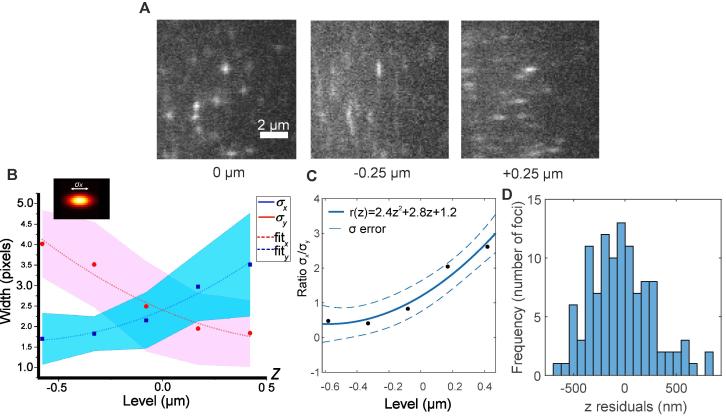
A: Micrographs of *in vitro* surface-immobilization assay, with purified GFP molecules bound to a glass coverslip surface. Here we illustrate offsets of −0.25 µm, 0 µm, and + 0.25 µm to show the deformation of the PSF image at different depths. B. Mean fitted x and y sigma values to *in vitro* GFP as a function of focal depth (blue and red squares), σ_x_ and σ_y_, with standard error indicated as shaded area and 2nd degree polynomial fits as dashed lines. C. The ratio of the fitted Gaussian σ_x_ to σ_y_ values as a function of focal depth (black squares) with 2nd degree polynomial fit was used to calculate z position and 1 sigma confidence interval values (full and dashed blue lines, respectively). Every point represents a mean value of σ_x_/σ_y_ ratio calculated from purified GFP molecules imaged in two datasets at five different levels: −0.5 µm (total 12 GFP spots analyzed), −0.25 µm (16 spots), 0 µm (symmetry level: 35 spots), +0.25 µm (27 spots), +0.5 µm (20 spots). (For interpretation of the references to color in this figure legend, the reader is referred to the web version of this article.)

To calibrate the fluorescent foci PSF image deformation due to the cylindrical lens, we imaged immobilized GFP on a coverslip at different axial distances, using a surface-immobilization assay adapted from earlier studies, where the presence of single-molecules was verified using single-step photobleaching [23], [24]. In brief, a ∼2–3 mm width channel chamber with a volume of ∼5 µl was created from two strips of a double-sided tape on a microscopy slide and covered with a plasma-treated BK7 coverslip. A PBS solution of 2 µg/ml anti-GFP antibody (Invitrogen, G10362) was flowed into the chamber and left to adhere to the coverslip surface for 5 min at RT. Excess antibody was washed away by 200 µl of PBS. Four chamber volumes of 1 µg/ml GFP were then injected into the chamber, left to conjugate with antibodies for 10 min, and washed to remove any unbound molecules. 300 nm diameter polystyrene beads (Invitrogen, C37281) in 1:1000 dilution were added to the slide to focus on the coverslip surface in the brightfield, before the nanostage was moved −150 nm to set the *z* = 0 position. Images of single immobile GFP molecules were acquired at a 4.7 ms exposure time at axial positions between *z* = −0.5 µm and *z* = +0.5 µm in 0.25 µm intervals (Fig. 2A) spaced at roughly point spread function widths apart and over a range consistent with the depth of field of the microscope. We used GFP *in vivo* rather than other labelling methods (such as SNAP-tag or HaloTag) to obviate any difficulties transfecting cells with a dye and labelling efficiency. Novel synthetic dyes (such as azetidine-substituted Janelia Fluor® fluorescent dyes) have higher quantum yields and are more photostable than fluorescent proteins but require electroporation to be introduced into the yeast cell [25], [26].

#### 2.2.2 Axial distance calibration curve

Images of immobilized GFP molecules were tracked using bespoke super-localization software written in MATLAB (MATHWORKS) [27], modified to fit each fluorescent foci PSF image using a standard 2D lateral Gaussian function, to obtain its sub-pixel centroid, with independent σ width parameters, σ_x_ and σ_y_. The mean σ_x_ and σ_y_ values were collated for each axial position and are shown in Fig. 2B, indicating similar results to other 3D microscopes [28]. Ultimate calibration of the ratio of sigma widths, *r = σ_x_/σ_y,_* to the axial position, z*,* was obtained by fitting an optimized 2nd order polynomial (Fig. 2C):

$$r\left(z\right)=2.4z^{2}+2.8z+1.2$$

which gave a goodness-of-fit parameter *R*^2^ = 0.96. The data can also be fitted by an explicit de-focusing equation if required [29] but the form of the fit is in practice not critical since model-dependent differences to fits in general are small compared to the actual empirical axial precision [30].

### 2.3 Simulation of 3D tracks

**Fig. 3:**
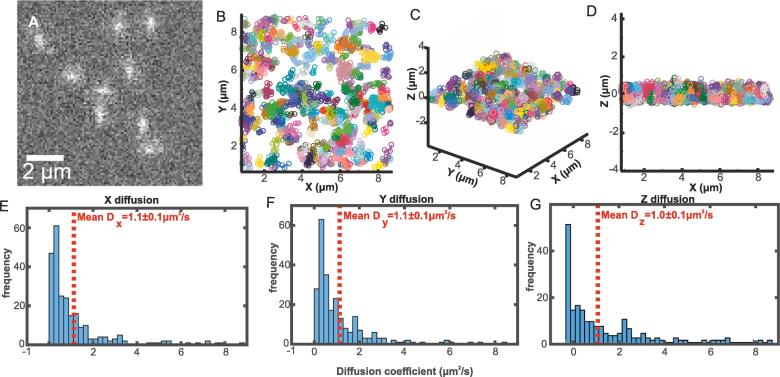
A, A single time sample image frame taken from a simulated sequence of fluorescently labelled molecules diffusing in three spatial dimensions. B-D, Scatter plots of tracked simulated diffusing molecules. E-G, Distribution of diffusion coefficients of tracked simulated data in all three spatial dimensions.

In order to verify that our astigmatism microscope could be used to track diffusing molecules as a function of time and verify our calibration, we simulated extended kinetic series of diffusing molecules whose fluorescence intensity was consistent with those measured experimentally, with realistic levels of added noise. We then measured their apparent microscopic diffusion coefficients using exactly the same detection and tracking analysis algorithms as for the experimental data. Astigmatism-deformed fluorescent foci were simulated by taking the average real mean foci image at each axial displacement, then linearly interpolating between each image at intervals of the equivalent camera pixel magnification which was 93 nm per pixel (SI Fig. 2A and B) to create a series of reference images spanning a 1 µm range in *z*, comparable to the approximate axial working range over which we could reliably detect single GFP molecules. 4D Foci positions (i.e. spatial coordinates for *x*,*y, z,* and also the time dimension *t*) were simulated using Brownian motion with a nominal diffusion coefficient of 1 µm^2^/s based on sensible experimental estimates from earlier 2D measurements [7]. The correct reference image was added to an array at each 4D position, and then realistic camera and signal noise were added to the array (we used a mean camera offset value of 100 counts with Poisson-distributed noise) (Fig. 3A). Images were tracked similarly for the *in vitro* calibration data (Fig. 3B–D), their mean square displacements (MSD) were calculated separately in each dimension (SI Fig. 2C), then their apparent microscopic diffusion coefficients were calculated from a linear fit to the first four MSD time interval values, constrained through the theoretical localization precision based on foci intensity [31] (Fig. 3E–G) using a previously optimized method [32].

### 2.4 Live cell microscopy

For live cell imaging, we used the model unicellular eukaryote of budding yeast *S. cerevisiae*, strain YML14, expressing genomically integrated Mig1-GFP (Mig1 is a transcription factor acting as a repressor for several target genes implicated in glucose metabolism) and Nrd1-mCherry (Nrd1 is a protein component of the RNA polymerase as is a clear marker for the position of the nucleus) fusions [7]. Cells were grown in minimal, transparent Yeast Nitrogen Base (YNB) media (1.7 g/l Yeast Nitrogen Base without amino acid and (NH_4_)_2_SO_4_, 5 g/l (NH_4_)_2_SO_4_, 0.79 g/l complete amino acid supplement as indicated by the manufacturer) supplemented with 4% glucose until mid-logarithmic growth phase, washed and placed into 3 ml of fresh medium for about 1 h. 5 µl of the culture was applied onto a 1% agarose pad perfused with YNB, formed using a 125 µl volume Gene Frame® (Thermo Scientific) and covered with a plasma-cleaned BK7 22 × 50 mm glass coverslip. Typically 1–4 cells per field of view were imaged using conditions similar to those described previously [7], [33].

We employ a non-sparse approach to single-molecule imaging. Unlike PALM and STORM where only single emitters are excited and imaged, we excite everything. By exciting all fluorophores at once and using step-wise photobleaching, we can quantify the stoichiometry and copy number of proteins in live cells dynamically, as in previous studies [7]. We applied this method to Mig1 and found that this protein forms clusters which are present on the DNA in the nucleus. Here we are able to track foci from the start of acquisitions, all the way through the photobleach until single-molecules of Mig1-GFP become visible, thus this method is applicable to clusters and single-molecules.

### 2.5 Analysis of live cell data

Images of Mig1-GFP (SI movie 1) were tracked using the same methods as before [7]. Frame averages were taken over five consecutive images of Mig1-GFP and Nrd1-mCherry, segmented for the cell and nucleus respectively using the GFP and mCherry signals respectively. These images were then thresholded, which created distinct masks for the nucleus, though often left multiple cells joined together in a single mask. To overcome this issue an extra watershedding step [34] using the nucleus masks as seed basins for the joined cell masks allowed true cell masks to be obtained of each separate cell. Mig1-GFP foci tracks were then assigned into cells and the separate sub-cellular compartments (i.e. cytoplasm or nucleus) based on their positions. Mig1-GFP tracks lasted up to 1 s with a mean track length ∼70 ms. The apparent microscopic diffusion coefficients of each foci track were calculated as in our previous 2D study but now using the full MSD determined from the complete 3D spatial localization data. We used the diffusion coefficient threshold defined in our previous study to collate the putative immobile Mig1 tracks as being those with a rate of diffusion at or below 0.1 µm^2^/s [7]. We then calculated the fluorescence intensity centroid of these tracks to define the position of immobile Mig1 in the nucleus, and thus a putative Mig1 binding site on the genomic DNA.

## 3 Results and discussion:

### 3.1 Calibration and performance of the microscope

By extrapolating the possible z range from the error in Fig. 2C, we estimate that our calibration yields an axial resolution of ∼100 nm, roughly 2–3 times poorer than our measured lateral resolution of ∼40 nm under comparable imaging conditions [31]. We also calculated a similar axial resolution of 106 ± 10 nm by tracking the *in vitro* calibration data and comparing the measured to the known axial distance. (SI Fig. 1C). In x and y we measured ∼90 nm resolution (SI Fig. 1A and B). This reduction in spatial resolution from lateral to axial is similar to other previously implemented 3D light microscopes [28] and compares favorably with other astigmatism based microscopes. Although others have reported superior axial resolution using astigmatism approaches, for example Huang [30] achieved 30 nm lateral resolution and 50 nm axial resolution using a 3D astigmatism STORM instrument imaging bright organic dyes. Fluorescent proteins probes are more challenging due to poorer photophysical properties, though Moerner reported axial precisions of ∼40 nm using yellow fluorescent protein by employing a double helix PSF method with 30 ms per frame sampling [35]. However, since this variant of YFP used emits approximately 175% more photons on average than GFP prior to photobleaching [36], and our method involves much faster sampling, also by close to an order of magnitude compared to Moerner’s YFP study, our reported axial precision is close to expectation based on the effective signal-to-noise ratio [37]. Our calibration using single GFP molecules also in many ways represents a worst case for axial resolution as many of the transcription factors we image are clustered [7]. New fluorescent proteins, such as mNeonGreen, are also ∼3x brighter [38] and will also increase the axial resolution by a factor roughly equivalent to the square root of the increase in brightness. Astigmatism also has advantages over double helix PSF microscopy: it is easier to implement in the microscope, it does not require permanent alignment of often expensive phase masks – allowing both standard widefield imaging and astigmatism imaging with a flip-in component, and the data analysis is simple.

Tracking simulated foci trajectories, we were able to measure the same apparent microscopic diffusion coefficients as simulated within expected sampling error in all three spatial dimensions. We calculated MSDs separately, in each dimension here to compare x and y diffusion obtained from standard tracking to z diffusion obtained from astigmatic PSF fitting, which should produce the same result if our method is correct. As they are the same, we are able to track molecules *in vivo*. Our measured diffusion coefficient distributions are broad but this is expected from the long tail of Gamma shaped probability functions [39]. Also, fitting to MSD *vs*. the time interval parameter to generate diffusion coefficients inherently generates positively skewed distributions as extreme high values are not detected due to the limits of tracking while errant low diffusion coefficients result from poor tracking. The latter is due to linking different foci incorrectly into the same trajectory, resulting in apparent reduction in MSD and fits which tend towards low values. Error can also be introduced from overlapping foci with multiple foci in close proximity being detected as one extended foci. All of these factors combine to broaden the measured diffusion coefficient distributions, but our simulations show that the correct population statistics can still be extracted.

### 3.2 3D architecture of Mig1 binding sites

**Fig. 4:**
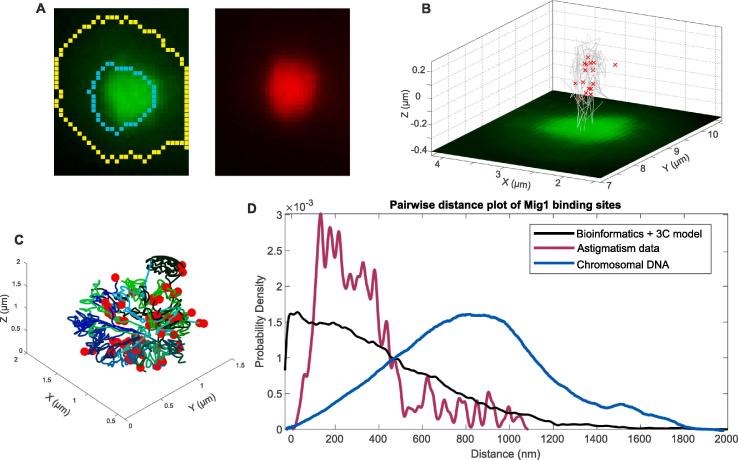
A. Fluorescence micrographs of Mig1-GFP (green) and Nrd1-mCherry (red) with segmentation for cell and nucleus (yellow and cyan) overlaid. B. Mig1-GFP in a single live yeast cell (green) with the 3D position of immobilized Mig1 molecules in the nucleus marked as red crosses and their trajectories indicated as grey lines. C. The 3C model of yeast chromosomal DNA (green and blue lines) from reference [40]) with the position of Mig1 binding sites within promoters marked as red dots from reference [7]). D. The distribution of pairwise distances of detected immobilized Mig1 foci in the nucleus (maroon), predicted from Mig1 binding sites in promoters in the 3C model (black) and from all chromosomal DNA positions in the 3C model. (For interpretation of the references to color in this figure legend, the reader is referred to the web version of this article.)

We took the 3D centroid positions of immobile Mig1 tracks as the putative positions of Mig1 binding sites in the genome and an indicator of 3D genome architecture (Fig. 4A and B and SI Fig. 2). We compared these positions to those obtained from a predictive model, using bioinformatics to map out likely Mig1 binding sites within promoters onto a 3C model of yeast chromosomal DNA (Fig. 4C) [7]. Potential Mig1 binding sites were identified by analyzing the whole genome of *S. cerevisiae* in order to find DNA sequences that fit a pattern Mig1 binding site motif generated using the UIPAB nucleotide code based on 14 well-characterized Mig1 target sequences [41]. To compare experimental and modeling outcomes, we calculated the distribution of pairwise distances of observed immobile Mig1 foci in 25 cells and of potential Mig1 binding sites in the model (Fig. 4D). The distribution from the astigmatism data is different to the theoretical prediction. The mean pairwise separation of the predicted distribution is 417 ± 30 nm, ±SE, compared against 330 ± 7 nm, with a Student’s *t*-test indicating different means (*P* < 0.0001), although if pairwise distances greater than our working 1 µm range are excluded from the predicted distribution, the mean is 368 ± 25 nm which although marginally closer to our experimental measurements is still statistically different (*P* < 0.0001).

This intriguing observation may result from several possibilities. For example, if only certain areas of the genome are undergoing active transcription, then it is possible that the range of the pairwise differences could be relatively higher than expected but that the peak value could potentially be smaller if the regions of active transcription are themselves relatively clustered. Similarly, the clustered nature of Mig1 implies multivalency of binding to DNA, and this in turn may result in condensation effect of separate DNA strands which are linked by a cluster, thus shifting the mean pairwise distance. It may also be the case that only a subset of spatially clustered Mig1-regulated genes have Mig1 bound to the target promoters at any one time: a subset of Mig1 clusters might also bind transiently (at least over a duration of four consecutive image frames or 20 ms used for the diffusion coefficient estimates), but in a relatively immobile state, to regions of the genome which are not specific promoter regions. Such a phenomenon could indeed be functionally important in intersegmental transfer of clusters between DNA segments [42]. This putative hopping motion may reduce the search time for Mig1 to ultimately find its gene targets, and thus might be expected to occur at regions which are not collocated with the Mig1 binding sites themselves. Hopping between separate DNA segments may of course result in transient immobility to the translocation along the original DNA segment prior to reaching the destination DNA segment.

Our 3D imaging approach has revealed significant tiers of complexity to the dynamic architecture of the genome, which slower, less precise and less physiologically relevant techniques would not be able to render. It remains to be determined in future studies precisely what are the key explanations for this heterogeneity in the genomic architecture, but our methodology shows promise in being able to enable such future insights. While the 3C model is an ensemble technique which represents an average conformation of a genome, our method reconstructs 3D genome architecture at a given time and thus potentially enables detailed studies of its dynamics in a single living cell.

### 3.3 Conclusions

Here we describe a method which enables us to map out the 3D positions of immobile fluorescent DNA-binding molecules, which thereby act as a valuable proxy to indicate the genome architecture in live yeast cells. We apply it to the Mig1 transcription factor for which we have a predicted model of the 3D position of binding sites within the genome from a knowledge of its binding sequences within the target gene promoters applied to prior 3C data. This allows us to provide more information towards understanding the functional 3D genome architecture in live cells. Recent articles, mainly based on ChIP-Seq technique, suggest that for many DNA binding proteins not only sequence specificity but also DNA shape defines target sites within the genome [43], [44]. However, studies on yeast transcription factors show that in many but not all cases, computational affinity predictions based on conservation motif discovery, agree with models created from experimental data [45], [46]. Therefore, determination of the exact target sites remains a challenging open question which could be resolved by direct observation of protein-DNA interactions in living cells. In our low signal to noise simulations, we were able to measure simulated diffusion coefficients within 10% error using MSD fitting, which we consider to be a good fit for single-molecule data. These fitting methods we developed previously [7], and were validated using a complementary ‘Jump Distance’ based analysis (see [47]). It should be noted that there are alternative methods to generate diffusion coefficients from MSD data reported by others, for example so-called vbSPT [48] and HMM-Bayes [49], featuring in a range of publications, e.g. [50], [51]), and both methods find that MSD fitting is a valid method provided the number of MSD points is optimized; too large and the effect of non-Brownian motion and fluorescent lifetime affect the result. Here, we restrict the MSD fitting to just four data points, as used in several previous recent studies [6], [7], [52], [53]. In [51], the authors also make valuable insights by highlighting the problems of too few MSD points due to the localization precision. Our approach here is to constrain the fit to MSD through the localization precision (the point of intercept on a plot of MSD *vs.* time interval relation) as calculated based on the signal to noise of the fluorophore [31], based on the theoretical precision [54]. However, an important point to note is that these alternative methods for extracting diffusion coefficient values do require well established prior knowledge (i.e. physical models), for example in Bayesian approaches these need to be incorporated explicitly into the prior function. It is non-trivial to achieve these prior functions for clusters of transcription factors in the nucleus when the mobility characteristics are relatively under-theorized at the very rapid millisecond sampling we use here. Our method instead is simpler and arguably of lower precision as a result, but in requiring fewer assumptions in regards to physical models is likely to be subject to less potential systematic bias and so we suggest is a sensible starting point at least in these types of analyses. Of course, this does not preclude the use of alternative methods for extracting diffusion coefficient values if sensible and well-characterized physical models concerning mobility behavior do indeed exist.

Recent methods now allow single-cell 3C to be combined with microscopy [55], although currently only using lateral imaging (i.e. 2D, in 2 spatial dimensions). If this technique were combined with our 3D method, it could potentially unlock transformative levels of information about genome architecture. Our method here is timely, given the increasingly revealed complexity in the dynamics of transcription factors and the impact of 3D DNA geometry on gene regulation [56], as well as other new methods using single-molecule FISH (smFISH) approaches which can map chromatin in 3D at a single-cell level [57], [58]. Our general method could in principle be extended to many other native proteins and even chromatin, or artificially expressed markers for specific genome loci in both eukaryotic and prokaryotic organisms. For example, tagging of the *Lac* operon of *Escherichia coli* [59], [60] could be used to report on the 3D architecture of highly specific sites within the prokaryotic genome. Although as it stands the spatial precision of our 3D imaging toolkit is poor compared to 3C variant approaches and smFISH and renders only snippets of the genome in snapshots, there is a substantive advantage in our approach in enabling us to interrogate these snippets of the 3D genomic architectures using very high time resolution of milliseconds on single live cells. A really exciting future of this technology could perhaps lie with combining this exceptional time resolution with a higher spatial resolution technology, such as 3C or smFISH based methods, as well as precise determination of protein binding sites within DNA.

## Funding sources

This work was supported by the European Commission via the Marie Curie Network for Initial Training ISOLATE (grant number 289995); the Biological Physical Sciences Institute (BPSI); a Royal Society Newton International Fellowship (grant number NF160208) and the Wellcome Trust (grant number 204829) through the Centre for Future Health at the University of York; and the BBSRC (grant numbers BB/P000746/1and BB/N006453/1.

## Declaration of Competing Interest

None.

## Data Availability

The full tracking and analysis code is available here https://github.com/awollman/single-molecule-tools.
