# Supplementary material for: Towards mapping the 3D genome through high speed single-molecule tracking of functional transcription factors in single living cells
Source: Methods. 2020 Jan 1;170:82–9. doi: 10.1016/j.ymeth.2019.06.021 (PMC6971689; doi:10.1016/j.ymeth.2019.06.021)
Supplement: Supplementary data 2 [file mmc2.pdf]

Supplementary Information

Towards mapping the 3D genome through high speed single-molecule tracking of functional transcription factors in single living cells

Adam J. M. Wollman[a,1], Erik G. Hedlund[b,1,2], Sviatlana Shashkova[c,1], Mark C. Leake[d]

[a] Biological Physical Science Institute, Departments of Physics and Biology, University of York, YO10 5DD York, UK (adam.wollman@york.ac.uk)

[b] Biological Physical Science Institute, Departments of Physics and Biology, University of York, YO10 5DD York, UK (erik.hedlund@kuleuven.be)

[c] Biological Physical Science Institute, Departments of Physics and Biology, University of York, YO10 5DD York, UK (sviatlana.shashkova@york.ac.uk)

[d] Biological Physical Science Institute, Departments of Physics and Biology, University of York, YO10 5DD York, UK (mark.leake@york.ac.uk)

[1] These authors wish it to be known that they have contributed jointly.

[2] Centre for Surface Chemistry and Catalysis, KU Leuven, Celestijnenlaan 200F - box 2461. 3001 Heverlee, Belgium (erik.hedlund@kuleuven.be).

Corresponding author: Mark C. Leake (mark.leake@york.ac.uk)

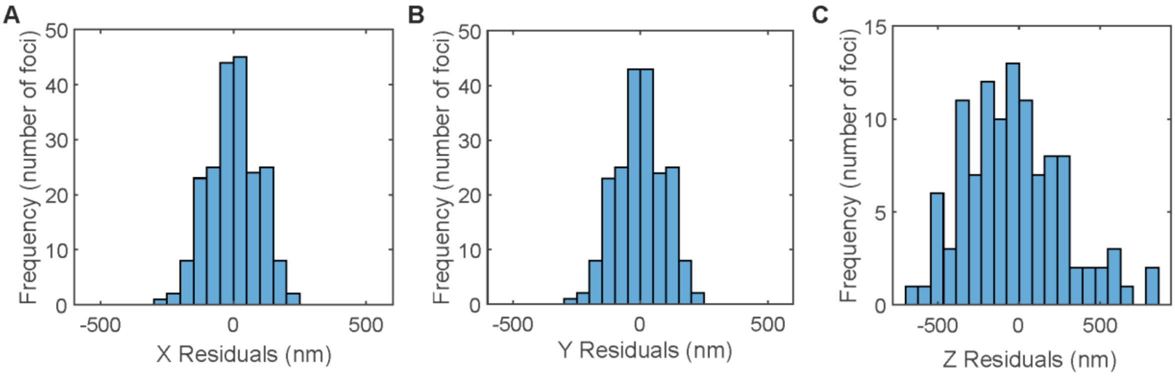

Supplementary Figure 1: A. and B. Histogram distributions of residual displacement from mean in vitro foci centroids. C. The residuals between tracked foci axial distance and the ground truth axial distance in in vitro calibration.

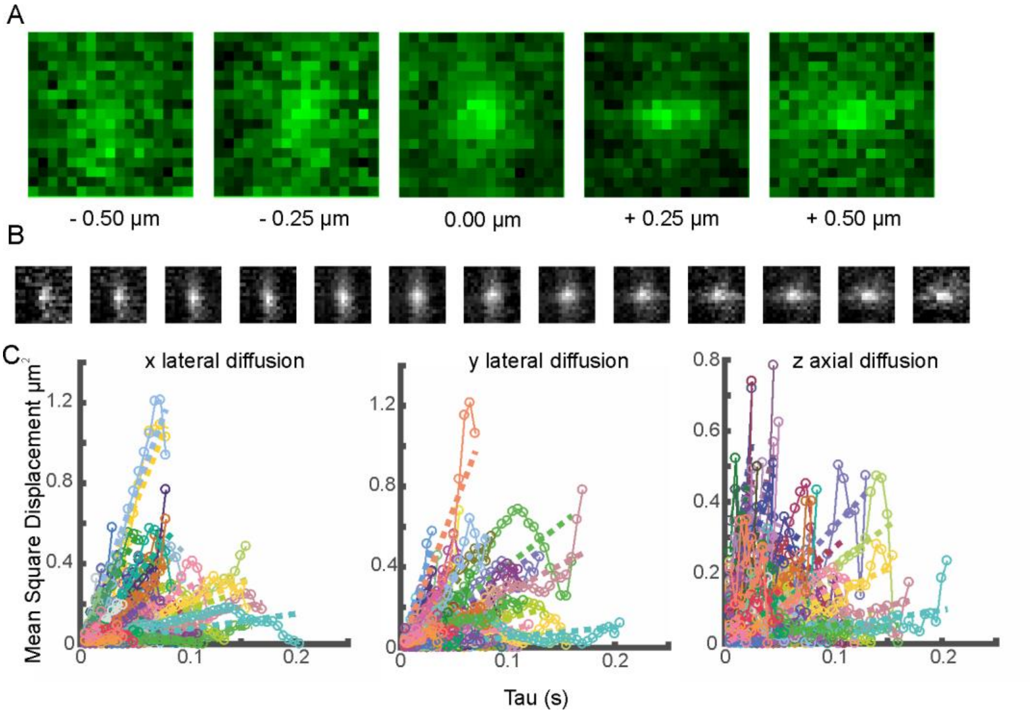

Supplementary Figure 2: A. Mean PSF as a function of axial distance z as based on in vitro *GFP images. B. Interpolated PSF as a function of z used in simulations. C. Mean square displacement vs. time interval (Tau) for simulated trajectories.*

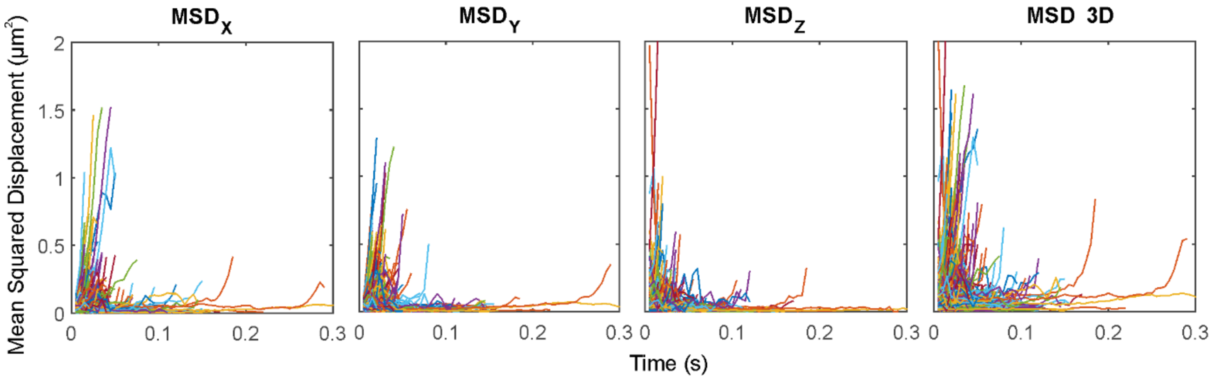

Supplementary Figure 3: Mean squared displacement as a function of time for in vivo *trajectories in x,y,z and 3D.*

Description	***Manufacturer***	***Part Number***
Dichroic mirror in the objective turret	Chroma	Di01–R488/561
Notch filter in the imaging path	Chroma	ZET473
300mm cylindrical lens	Thorlabs	LJ1996L1-A
Dichroic mirror in the DV2 color splitter	Chroma	565dcxr
Emission filter in DV2 color splitter	Chroma	D520/30M
Emission filter in DV2 color splitter	Chroma	D630/50M

Supplementary Table 1: Filters and cylindrical lenses used in single-molecule microscope

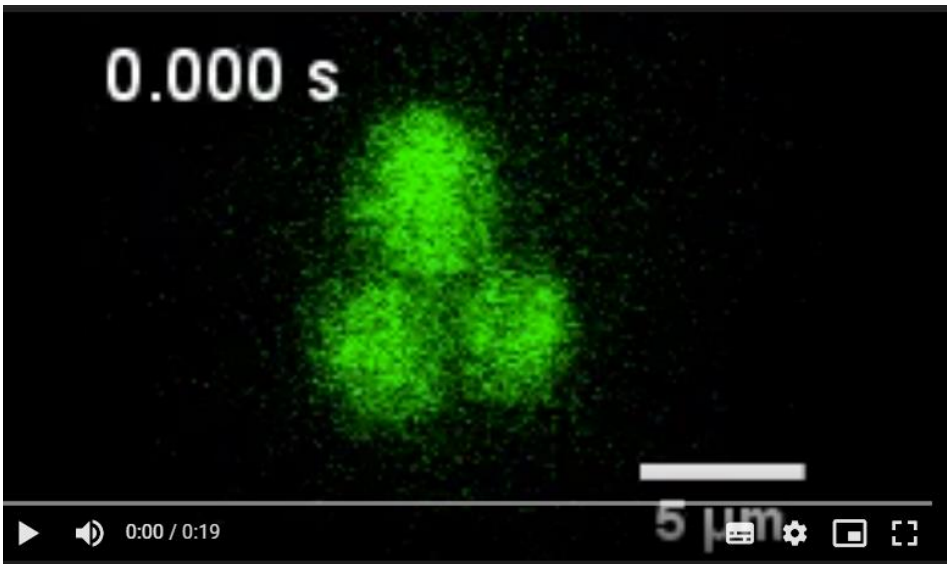

Supplementary Movie 1: *Astigmatism imaging Mig1 foci in live yeast cells.*
